# Spillover Effect of the Internet on Trade Performance Based on a Vision of the Public's Sleep Health: A Spatial Study of the Global Network

**DOI:** 10.3389/fpubh.2021.806694

**Published:** 2022-01-13

**Authors:** Xiaotao Zhang, Da Huo, Shuang Meng, Junhang Li, Zhicheng Cai

**Affiliations:** School of International Trade and Economics, Central University of Finance and Economics, Beijing, China

**Keywords:** internet spillover effect, sleep health, trade performance, spatial modeling, global network

## Abstract

This is the first study to analyze the spatial spillover effect of the internet on trade performance based on a vision of the public's sleep health. The internet's effect on trade performance has been enhanced in a new economy consisting of larger global markets. An overall improvement in health gradually impacts economic development. In this study, hierarchical modeling is applied to reveal the effect of the internet on trade performance at a fundamental level, and the effect of sleep health on trade performance at general level. The global network is structured by a spatial weight matrix based on the Mahalanobis distance of the internet and sleep health. Furthermore, spatial autoregressive modeling is applied to study the effect of the spatial weight matrix based on the Mahalanobis distance matrix of the internet and sleep health on trade performance. The spatial Durbin modeling is applied to further analyze the interaction effect of the spatial weight matrix and countries' factors on trade performance. It was found that the internet has a positive effect on trade performance, and good sleep health can be helpful to the spillover effect of the internet on trade performance. The interaction of the spatial weight matrix and gross domestic product (GDP) can further enhance the effect. This research can assist global managers to further understand the spatial spillover effect of the internet on trade performance based on a vision of the public's sleep health.

## Introduction

Since the birth of internet technology in 1969, the global economic situation has been gradually influenced by it. The digitalization of economic procedures and its endowment of knowledge elements makes the development of the internet a key component of economic research. The development of the internet has greatly reduced distance on the physical level within the scope of abstraction, making information dissemination more rapid and information processing more efficient. International trade is changes constantly with the internet's development, which has influenced the direction, structure, and methods of trade such as the extension of trading scope or the iteration of trading mode. In recent years, countries worldwide have been vigorously promoting the construction of information infrastructure to realize the internet's dividend as soon as possible. The concept of internet-plus has taken root in various industries, and the boundaries of traditional industries have gradually become blurred. This has accelerated the cross-border flow of various resources, especially of information, and has effectively promoted the development of international trade and investment.

As societies shift from an information economy to an internet economy, Organization for Economic Co-operation and Development (OECD) countries are strengthening their strategic approaches to digital transformation, according to the OECD 2020 Digital Economy Outlook. Several countries take internet development as a new driving force for economic growth as they face the reality of declining economic growth, adjust their industrial structures and transform their economic growth mode. The expansion of the internet is considered to provide huge potential power for the occurrence of international trade.

Sleep health has become an increasingly prominent issue relative to the economy. With the boom in internet-based business platforms and mobile payment systems, its convenience for international trade is not only reflected in changing the transaction place, saving the distance cost, and breaking the physical space limitation of traditional business activities. It is also reflected in breakthroughs in time and improved connection in global trade timelines. Merchants in different time zones can use the internet to conduct instant communication to extend the transaction window and improve the transaction speed, so that the trade activities are no longer limited to the day but are gradually extended to the night. Therefore, the amount of time people spend sleeping, has become a key factor to measure. Sleep deprivation can affect economic activities, national economies (1) and is costly to the economies in different locations. The sleep status of employees is especially important at an enterprise level, as employees' sleep deprivation affects enterprise performance by affecting work efficiency and creativity levels. Consequently, sleep is becoming increasingly important in a global economy.

In the context of rapid internet growth, international trade is also expanding due to reductions in transaction costs, the expansion of transaction markets and other factors. The advancement of information technology further accelerates economic development (2). Moreover, the internet did more than just break the spatial limits of the market. The global network is further structured based on enhanced information flow within the digital economy (3). Geographic differences also have a significant role in the development of the economy (4). Global timelines are no longer independent, and the amount of time people in different time zones spend at work and at rest, correlate. Therefore, it is necessary to study the effect of the internet on international trade using a network and spatial model (5, 6). To some extent, sleep has become a crucial factor in the study of the internet and international trade. Therefore, this paper will further explore the role of sleep factors in the relationship between the internet and international trade.

Our research makes three contributions to the literature. This is the first study to analyze the spatial spillover effect of the internet on international trade performance based on a vision of the public's sleep health, which has been a research gap. The application of a spatial weight matrix based on the Mahalanobis distance offer support to further study the spillover effect for economic development in the global network (7). Spatial autoregressive modeling and spatial Durbin modeling which aim to study the economic spillover effects, were applied. The conclusions have implications for policy makers and global managers.

This research contributes to current literature in explaining the spillover effect of internet on trade performance. The digital economy facilitated by development of internet offers an important driving power to new growth, and the literature in digital economy is looking for a further exploration to the unique effective mechanism of internet on trade performance. The public sleep health can be an initiative vision generated from public health behavior in internet uses, and offers an important explanation to the spillover effect of internet on trade performance based on interconnections in life traditions. The similarity of public sleep health can structure solid interconnections across different country nodes in global network, and enhances the externality effect of internet on trade performance.

## Literature Review

### Effect of the Internet on Trade Performance

Most prior studies show that the vigorous development of the internet has a positive impact on economic growth. The panel data of countries was studied as samples and the macro production function was incorporated into the micro model. The information flow accelerated by internet can be an important support to the new growth in economic development (8). The positive effect of internet on trade performance can be further enhanced by two-sided platform mechanism (9). The internet infrastructure construction of a country is beneficial to its economic growth (10). Furthermore, internet infrastructure impacts on economic development may have endogenous problems (11). Internet infrastructure is conducive to economic growth. The cross-border e-business facilitated by the internet has further enhanced trade performance (12).

Internet infrastructure ought to be distinguished from other infrastructure such as transportation and electricity but as a representative of national informatization levels and technological progress. The level of internet development affects enterprises' productivity. The construction of internet infrastructure and the popularization of the internet indicates a benign mechanism of informatization through both traditional and network paths (13). Traditional path means that the internet, as information technology, is applied in enterprise production and operation to directly improve enterprise productivity. Network path means that the technology spillover and diffusion of the internet contributes to total factor productivity through network effect. The development of infrastructure offers further support of the spillover effect in economic development (14). Internet infrastructure is used as a proxy for technology level and is scientific and technological. The vigorous development of the internet can promote economic growth in various ways (15).

The construction of the internet can have a positive impact on international trade by promoting industrial development, structural adjustment, and industrial upgrading. Internet technology promoted international trade by reducing transaction costs and broadening trade markets (16). The ecosystem-specific advantage offers further support to companies involved in internet economy based on externality effect (17). Progress in internet development is conducive to the quality and basic service level of banks (18). The construction of internet infrastructure and the popularization of the internet had a positive correlation with agricultural trade (19). At the country level, the development of the internet has a positive effect on service trade (20). Internet infrastructure construction has positive trade effects, especially for less developed countries. Web host numbers are used to represent internet popularity and to empirically test its impact on trade (21). The results indicate that along with an increase in internet penetration, exports also increase; an increase in the number of web hosts can increase the country's export by about two percentage points.

Information technology can facilitate trade by reducing exporters' fixed costs such as access to information, establishing distribution channels, advertising, and other transaction costs (22). At the national level, the cost of searching for information between importing and exporting countries will reduce as the internet improves (23). Therefore, efficiency in decision-making in international trade will improve. On the other hand, at the enterprise level, the construction of the internet can increase its advantages in international trade by reducing the cost of production, logistics, organizational management and communication with suppliers and consumers (24). The internet's development level is closely related to the scale and quality of international trade. Enhancing the development of the internet is conducive to upgrading foreign trade and stimulates international trade.

H1: The internet has a positive effect on trade performance in global network.

### Sleep Health and the Internet's Effect on Trade Performance

Sleep duration is closely related to business management and performance. Sleep deprivation would significantly negatively affect employees' work performance at the individual level through physiological, emotional, cognitive, and behavioral aspects (25). A healthy organizational culture should ensure that employees sleep well to improve their work output according to self-depletion theory (26). On the other hand, social resource conservation theory explains the relationship between employee sleep quality and performance (27). Sleep quality also has an impact on employee creativity fluctuations. Insomnia negatively affects creativity by inhibiting divergent thinking activities (28). Sleep quality negatively affects employees' job performance on a cognitive level (29). Insomnia also reduces performance by affecting employees' physical and mental health (30).

As an important factor to ensure the physical and mental health of employees, sleep can play a significant role in economic activities by improving the quality of enterprise human capital. The realization of individual functions has a significant relationship with health status, indicating that individual physical and mental health is a key factor to measure overall human capital levels (31). Health is an important indicator to measure human capital (32). Employee health, as the second stage of organizational health, gradually plays an increasingly vital role as one of the important assets in the process of creating value for enterprises (33). Investment in healthy human capital can effectively stimulate long term economic growth (34). Human health capital can increase the return rate of individual education investment by improving individual life expectancy, and improvements in health levels can reduce the depreciation rate of human capital (35). An organizational culture that supports employees' work-life balance and thus enables them to have more positive psychological levels will have a significant positive effect on employees' well-being (36).

The internet spillover effect plays a vital role in international trade. The development of information technology has significant influences on spatial organization structure and economic development relationship between cities within countries (37). The differences across difference countries can be important to business performance (38). The behavioral and cognitive differences in social life have influences to the economic performance in institutional environment (39). Innovation spillovers arise due to mutual influence between regions in the cross-regional flow of talent and knowledge and trade (40). The global network can have an important function in economic development across different countries (41). In addition, geographical distance affects the degree of innovation spillover, and the shorter the distance, the more obvious the spillover effect. Several factors affect internet spillover. The internet can produce spillover effects on the regional economy through three paths: economy, proximity, and urbanization (42).

Different sleeping habits may impact the way the internet is used and its effectiveness. Technology use is intricately linked to people's work-life balance (43). Sleep levels affect people's cognitive levels (44). Personal perception factors have a significant impact on internet use intentions (45). Current health issues and access to information skills are particularly influential in individuals' internet use (46). Therefore, sleep, as an important indicator of health, has an impact on the use of the internet. Human capital has a significant positive impact on technology adoption (47).

Sleep may impact the relationship between the internet and international trade. Human capital gradually plays a more vital role in the vigorous development of the internet (48). The degree of human capital investment in host countries would significantly affect technology spillover (49). The human capital factor at the enterprise level relates closely to the internet factor in Spain (50). Users' well-being significantly improves the willingness to use the IoT (Internet of Things) which means happiness has a positive moderating effect on the willingness of internet users to use the IoT (51). Previous literature reveals the moderating effect of human capital structure on the internet's impact on total factor productivity (TFP). Improvement in management and human capital levels will strengthen the positive impact of information technology on TFP (52). Therefore, in an era of the internet's rapid development, the role of healthy and dynamic human capital in economic activities, including international trade, is becoming increasingly important.

Through past literature, we found that sleep can influence internet use by influencing people's health quality, happiness levels, and other life aspects. Therefore, good sleep health can benefit the spillover effect of the internet on trade performance.

H2: Sleep health has a positive effect on the internet's spillover on trade performance.

## Research Method

This study aimed to analyze the effect of the internet on trade performance based on a vision of the public's sleep health. Hierarchical modeling was applied to analyze the effect of the internet and sleep health on trade performance, with the effect of the internet on trade performance as the fundamental level and the effect of sleep health on trade performance as the general level. The effect of the internet's interaction with the exchange rate, GDP, and population on trade performance were also analyzed.

Furthermore, a spatial study of the effect of the internet and sleep health on trade performance was developed. The network of countries is structured by the Mahalanobis distance of countries based on the internet and sleep health. The Mahalanobis distance is measured as follows:


$$\begin{array}{l} d_{ij}=\sqrt{{\left(X_{i}-X_{j}\right)}^{T}\sum^{-1}\left(X_{i}-X_{j}\right)} \end{array}$$

X represents the dimension of countries by internet and sleep health. The application of the Mahalanobis distance in structuring the network of countries can be helpful to overcome the scale differences across different dimensions. The network structured by a spatial weight matrix based on the Mahalanobis distance of the internet and sleep health was also visualized.

The spatial weight matrix based on the Mahalanobis distance of the internet and sleep health was further applied in the spatial autoregressive model to study the spillover effect of the internet on trade performance based on a vision of sleep health. The spatial Durbin model was further applied to analyze the interaction effect of the spatial weight matrix and country factors.

The sample of this research includes Australia, Austria, Belgium, Canada, China, Denmark, Estonia, Finland, France, Germany, Greece, Hungary, India, Ireland, Italy, Japan, South Korea, Latvia, Lithuania, Luxembourg, Mexico, Netherlands, New Zealand, Norway, Poland, Portugal, Slovenia, South Africa, Spain, Sweden, Turkey, the UK, and the USA. The data sources span 2010–2019. The internet was represented by the server uses based on data sources from the World Bank. Sleep health is represented by the number of minutes spent sleeping daily from the OECD database. The export and import trade performance, and the GDP were obtained from the World Bank. Furthermore, the exchange rate was obtained from the International Monetary Fund, and the population was obtained from United Nations data sources and government reports.

## Results

Table 1 shows the result of hierarchical modeling through analysis of the effect of the internet and sleep health on the trade performance of countries. The fundamental level of the hierarchical model tests the effect of the internet on trade performance, and its general level tests the hierarchical effect of sleep health on trade performance by countries. Models 1 and 2 show that the internet has positive effects on export and import trade performance, and the GDP also shows positive effects on export and import trade performance. However, the relationship between sleep health and trade performance was not identified. In addition, Model 3 found that the exchange rate has a positive effect on export trade performance, and a negative effect on export trade performance by interaction with the internet. Additionally, population has a negative effect on export trade performance by interaction with the internet. Furthermore, Model 4 found that the GDP has a positive effect on export trade performance by interaction with the internet.

**Table 1:** Hierarchical model to effect of internet and sleeping health on trade performance.

	**Model 1 (Export)**	**Model 2 (Import)**	**Model 3 (Export)**	**Model 4 (Import)**
Server	0.022^***^	0.014^***^	0.097^*^	−0.016
Exchange	0.003	−0.014	0.083^***^	0.03
GDP	0.735^***^	0.769^***^	0.775^***^	0.731^***^
Population	−0.047	−0.023	−0.032	0.061
Server*Exchange			−0.006^***^	−0.002
Server*GDP			0.001	0.01^**^
Server^*^Population			−0.006^*^	−0.015^***^
Sleep	−1.323	−1.065	−0.708	−0.099
N	330	330	330	330

* *p < 0.05*,

** *p < 0.01*,

*** *p < 0.005*.

**Figure 1:**
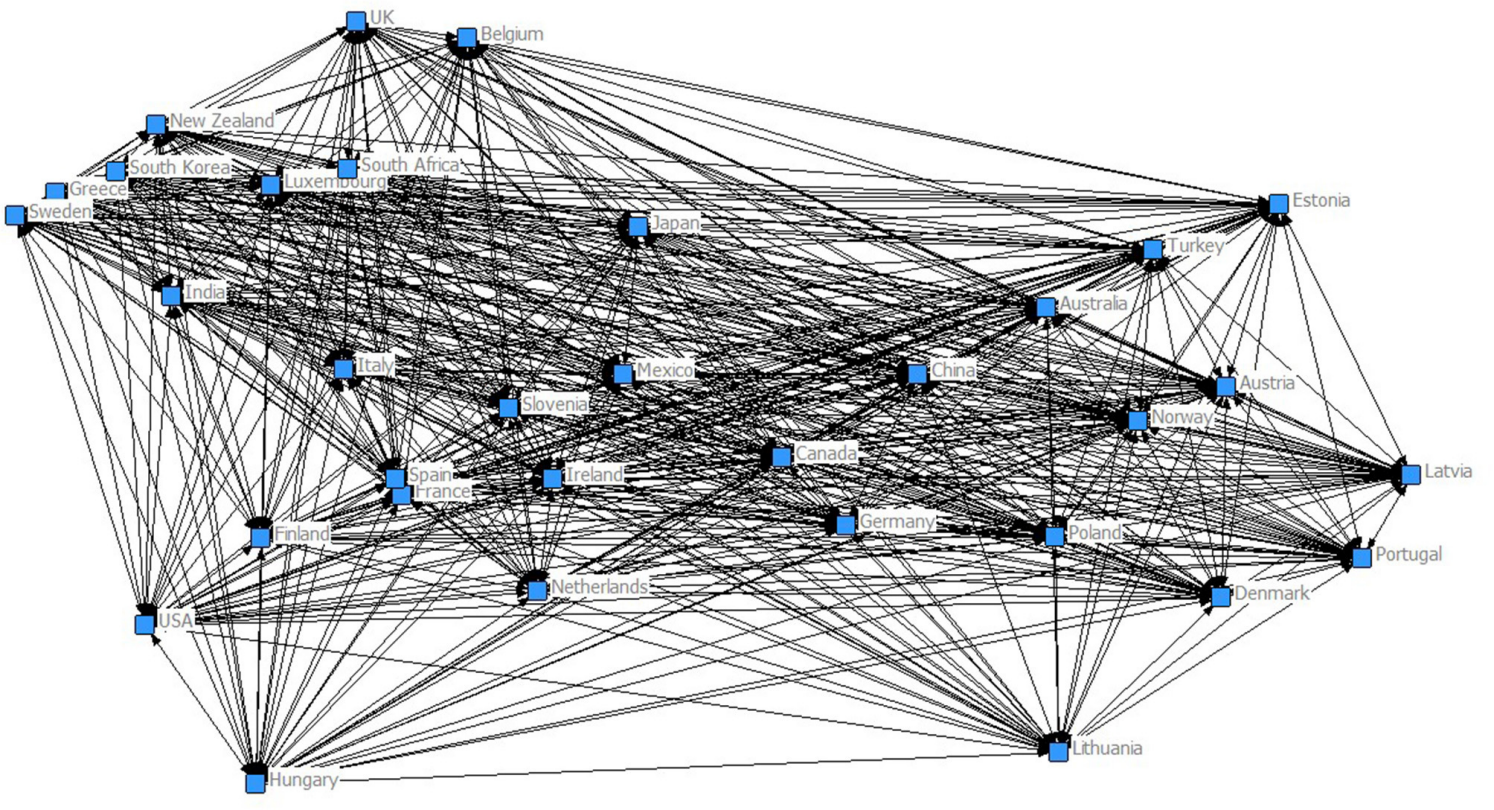
Global network structured by internet and public's sleep health.

Table 2 shows the result of spatial autoregressive modeling through analysis of the effect of the internet and sleep health on the trade performance of countries. The weight matrix in spatial modeling was structured by the Mahalanobis distance of countries based on the internet and sleep health. Figure 1 shows the global network structured by the Mahalanobis distance based on the internet and public's sleep health. The spatial spillover effect of the internet and sleep health on trade performance can be further analyzed by autoregressive spatial modeling. Models 5 and 6 found that the spatial weight matrix based on the internet and sleep health have both positive effects on export and import trade performance, with a significant positive effect of rho. The spatial spillover effect of the internet and sleep health on trade performance is supported. Similarly, good sleep health can be helpful for countries to work with the positive spatial spillover effect of the internet on trade performance. Good sleep health offers support to the spatial spillover effect of the internet on export and import trade performance. Models 7 and 8 further support this result. Moreover, there is support that the GDP has positive effects on both export and import trade performance. Model 7 further found that the exchange rate has a negative effect on export trade performance by interaction with the internet.

**Table 2:** Spatial autoregressive model to effect of internet and sleeping health on trade performance.

	**Model 5 (Export)**	**Model 6 (Import)**	**Model 7 (Export)**	**Model 8 (Import)**
Server	0.006	0.0002	0.141	0.026
Exchange	0.009	−0.009	0.092	0.039
GDP	0.639^***^	0.665^***^	0.719^***^	0.67^***^
Population	0.028	0.076	0.014	0.126
Server^*^Exchange			−0.007^*^	−0.003
Server^*^GDP			−0.004	0.006
Server^*^Population			−0.001	−0.01
rho	0.481^***^	0.502^***^	0.482^***^	0.473^***^
N	330	330	330	330

* *p < 0.05*,

** *p < 0.01*,

*** *p < 0.005*.

Table 3 illustrates the result of spatial Durbin modeling analysis of the effect of the interaction of the spatial weight matrix and country factors on trade performance. Models 9 and 10 found that the interaction of the spatial weight matrix based on the internet and sleep health and the GDP has positive effects on both export and import trade performance. It has been found that good sleep health can be helpful to the positive spillover effect of the internet on trade performance. This mechanism can be further enhanced by the interaction of the spatial weight matrix based on the internet and sleep health and the GDP. The negative interaction effects of the internet and exchange rate is further supported in Model 11, and the positive interaction effects of the internet and GDP is supported in Model 12.

**Table 3:** Spatial durbin model to effect of internet and sleeping health on trade performance.

	**Model 9 (Export)**	**Model 10 (Import)**	**Model 11 (Export)**	**Model 12 (Import)**
Server	0.022	−0.008	−0.021	−0.113
Exchange	0.006	−0.134	0.085	0.022
GDP	0.615^***^	0.669^***^	0.611^***^	0.593^***^
Population	0.111	0.082	0.093	0.134
Server*Exchange			−0.006^**^	−0.002
Server^*^GDP			0.003	0.01^*^
Server^*^Population			−0.001	−0.008
W^*^Server	−0.061	0.019	−1.262	−1.027
W^*^Exchange	−0.169	−0.162	−0.189	−0.648
W^*^GDP	0.369^*^	0.371^*^	0.717	0.828
W^*^Population	1.113	0.033	−0.668	−1.468
W^*^(Server^*^Exch)			−0.016	0.026
W^*^(Server^*^GDP)			−0.056	−0.067
W^*^(Server^*^Pop)			0.162	0.165
rho	0.167	0.146	0.218	0.153
N	330	330	330	330

* *p < 0.05*,

** *p < 0.01*,

*** *p < 0.005*.

## Discussion

Our research makes three contributions to the literature. This is the first study to analyze the spatial spillover effect of the internet on international trade performance based on a vision of the public's sleep health, which has been a research gap. The application of the spatial weight matrix based on the Mahalanobis distance offers support to further study the spillover effect for economic development in the global network. The spatial autoregressive modeling and spatial Durbin modeling which aimed to study the economic spillover effects were applied. The conclusions have implications for policy makers and global managers. It was found that the internet has a positive effect on trade performance, and good sleep health can be helpful for the spillover effect of the internet on trade performance. Besides, the interaction of the spatial weight matrix and gross domestic product (GDP) can further enhance the effect.

This study examined the spillover effect of the internet on trade performance based on a vision of the public's sleep health. Hierarchical modeling was applied to analyze the effect of the internet on trade performance on a fundamental level and the effect of sleep health on trade performance on a general level. The internet was found to have a positive effect on both export and import trade performance. Furthermore, the global network was structured by the spatial weight matrix based on the Mahalanobis distance of the internet and sleep health. The spatial autoregressive modeling based on the spatial matrix of the internet and sleep health was applied to study the spillover effect of the internet on trade performance based on a vision of sleep health. Good sleep health can be helpful to the spillover effect of the internet on both export and import trade performance. Furthermore, the spatial Durbin modeling was applied and found that the interaction of the spatial weight matrix based on the internet, sleep health, and the GDP enhance the spillover effect on trade performance.

This research contributes to the study of the economic effect of the internet on global markets based on a vision of public health, and it is helpful to global managers to further understand the internet's spatial spillover effect on trade performance based on a vision of the public's sleep health. This research launches a new vision to sociological effect of internet economy based on physiological characteristics in public's sleep health. The involvement of global network offers support to further study of new growth in geographic economy. The application of network science in spatial study highlights the research to spillover effect of internet on trade performance by support of similarity in public's sleep health, and offers further support to health economics in acquiring health science as an explanation to rationales in social economics.

This research offers an initiative support in explanation to effective channels of internet on trade performance by vision of life tradition based on public sleep health. Current literature has been intensively involved in revealing the unique effective mechanism of internet on trade performance, and the externality effects of internet in new growth driven by digital economy is a valuable contribution by internet on economic development. This research offers a new vision to current literature in digital economy, and empirically suggests that similarity of public sleep health offers a newly identified support to spillover effect of internet on trade performance. The viewpoint generated from research of public sleep health issue in traditional life behavior has shown a critical value in discovering unique explanation to externality effect of digital economy. The involvement of network science in spatial study of global network also leads a cutting-edge functional study in analyzing the economic issues from a public health vision.

## Data Availability Statement

The original contributions presented in the study are included in the article/supplementary material, further inquiries can be directed to the corresponding author.

## Author Contributions

XZ: conceptualization, investigation, validation, formal analysis, project administration, and writing review and editing. DH: conceptualization, resources, data curation, validation, methodology, formal analysis, project administration, writing–original draft, and visualization. SM: conceptualization, investigation, validation, formal analysis, and writing–review and editing. JL: conceptualization, formal analysis, and writing–review and editing. ZC: conceptualization, investigation, and resources. All authors contributed to the article and approved the submitted version.

## Funding

This research was supported by National Social Science Foundation of China General Project (20BJL055).

## Conflict of Interest

The authors declare that the research was conducted in the absence of any commercial or financial relationships that could be construed as a potential conflict of interest.

## Publisher's Note

All claims expressed in this article are solely those of the authors and do not necessarily represent those of their affiliated organizations, or those of the publisher, the editors and the reviewers. Any product that may be evaluated in this article, or claim that may be made by its manufacturer, is not guaranteed or endorsed by the publisher.
